# A homozygous missense variant in the alkaline phosphatase gene *ALPL* is associated with a severe form of canine hypophosphatasia

**DOI:** 10.1038/s41598-018-37801-2

**Published:** 2019-01-30

**Authors:** Kaisa Kyöstilä, Pernilla Syrjä, Anu K. Lappalainen, Meharji Arumilli, Sruthi Hundi, Veera Karkamo, Ranno Viitmaa, Marjo K. Hytönen, Hannes Lohi

**Affiliations:** 1 Department of Veterinary Biosciences, University of Helsinki, 00014 Helsinki, Finland; 2 Research Programs Unit, Molecular Neurology, University of Helsinki, 00014 Helsinki, Finland; 3 Department of Molecular Genetics, Folkhälsan Institute of Genetics, 00290 Helsinki, Finland; 4 Department of Equine and Small Animal Medicine, University of Helsinki, 00014 Helsinki, Finland; 5 Veterinary Bacteriology and Pathology Research Unit, Finnish Food Safety Authority Evira, 00790 Helsinki, Finland

## Abstract

Inherited skeletal disorders affect both humans and animals. In the current study, we have performed series of clinical, pathological and genetic examinations to characterize a previously unreported skeletal disease in the Karelian Bear Dog (KBD) breed. The disease was recognized in seven KBD puppies with a variable presentation of skeletal hypomineralization, growth retardation, seizures and movement difficulties. Exome sequencing of one affected dog revealed a homozygous missense variant (c.1301T > G; p.V434G) in the tissue non-specific alkaline phosphatase gene, *ALPL*. The identified recessive variant showed full segregation with the disease in a cohort of 509 KBDs with a carrier frequency of 0.17 and was absent from 303 dogs from control breeds. In humans, recessive and dominant *ALPL* mutations cause hypophosphatasia (HPP), a metabolic bone disease with highly heterogeneous clinical manifestations, ranging from lethal perinatal hypomineralization to a relatively mild dental disease. Our study reports the first naturally occurring HPP in animals, resembling the human infantile form. The canine HPP model may serve as a preclinical model while a genetic test will assist in breeding programs.

## Introduction

The genetic skeletal diseases are a group of inherited conditions that affect the bone and cartilage tissues. Although they are individually rare, their collective impact on welfare is significant, both in man and animals. In humans, the most recent update on genetic skeletal disorders encompasses more than 400 distinct conditions, resulting from defects in over 350 different genes^1^. One such condition is hypophosphatasia (HPP), a metabolic bone disease characterized by defective skeletal mineralization^2,3^.

HPP is caused by mutations in the alkaline phosphatase gene *ALPL*, which encodes an alkaline phosphatase isozyme, liver/bone/kidney-type, commonly referred to as tissue non-specific alkaline phosphatase TNSALP (alternatively TNALP or TNAP)^4,5^. The alkaline phosphatases (ALPs, phosphate-monoester phosphohydrolase, alkaline optimum; EC 3.1.3.1) constitute a family of metalloenzymes that in tissues localize to the cell’s outer membrane, where they dephosphorylate a variety of substrates, releasing inorganic phosphate (Pi)^6,7^. Today, the number of HPP-associated *ALPL* variants in humans has reached 370 (http://www.sesep.uvsq.fr/03_hypo_mutations.php), and the disease phenotype has also been recapitulated in mouse models^8–11^. The majority of human HPP patients are compound heterozygotes with a unique causative genotype, however, the disease can also be inherited in an autosomal dominant fashion, which is more frequent in milder disease forms and is associated with incomplete penetrance^12^.

The allelic heterogeneity of human HPP is reflected on the clinical spectrum, which ranges from stillbirth to a mild disease of the adulthood^12^. The disease is typically classified (in decreasing order of severity), into perinatal, infantile, childhood and adult onset forms, as well as into odonto-HPP, which is the mildest subtype affecting only teeth^13–16^. The major clinical characteristic of HPP is hypomineralization of bone and teeth, which results in defective ossification, osteomalacia and premature loss of dentition^3,17^. However, patients can also suffer from several accompanying clinical complications, such as respiratory failure, seizures, short stature, hypotonia, fractures and musculoskeletal pain^14,15^. The perinatal form corresponds to the most severe clinical picture with profound skeletal hypomineralization and the poorest prognosis^15,18,19^. In recent years, enzyme replacement therapy, using a recombinant TNSALP protein (asfotase alfa), has given promising results in the treatment of HPP patients^20–22^.

Purebred dogs (*Canis lupus familiaris*) are affected with a high number of naturally occurring genetic disorders that are often breed-specific or found only in a few related breeds. This is a consequence of historic population bottlenecks, inbreeding and genetic isolation of breeds^23,24^. Regarding inherited skeletal disorders, genetic studies in dogs have revealed shared disease genes with humans^25–28^ as well as novel gene-phenotype associations^29,30^. In the present study, we aimed to elucidate the clinical, pathological and genetic aspects of an inherited skeletal disease found in the Karelian Bear Dog (KBD) breed. The KBD is a Finnish primitive-type hunting dog that originates from the Karelia region of present-day Russia and is still actively used to hunt large game. We have previously recognized another genetic skeletal disorder in KBDs, a disproportionate chondrodysplastic dwarfism, caused by a homozygous nonsense variant in the integrin gene *ITGA10*^29^. In the current work, we were able to define the second skeletal phenotype in the breed as HPP, which now represents the first report of naturally occurring HPP in animals.

## Results

### Clinical and radiographic examinations reveal a severe skeletal disease

**Figure 1:**
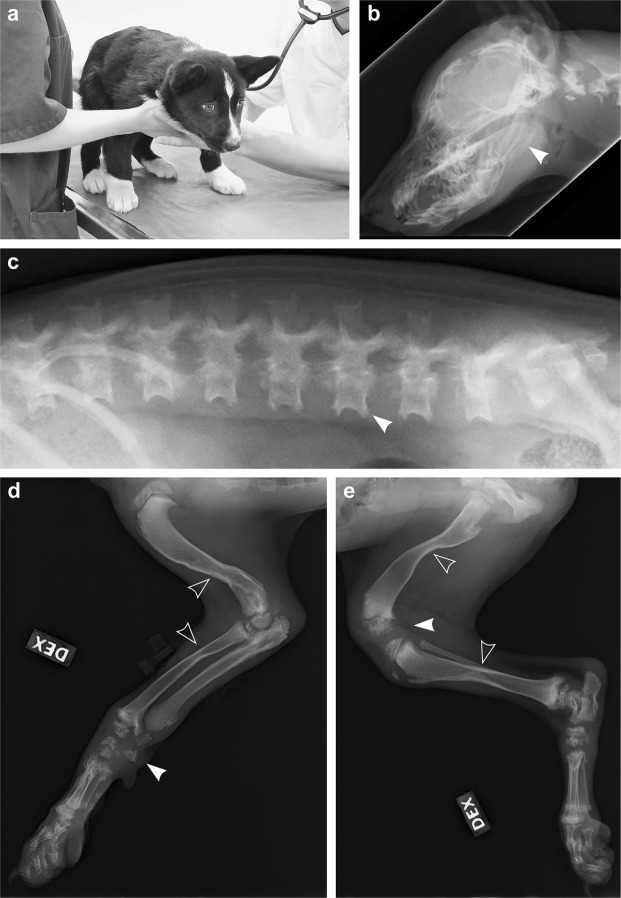
Radiographic findings in a 3-month-old affected dog. **(a)** The oldest affected dog (litter 1) had a crouched, planti- and palmigrade stance and gait. **(b)** Laterolateral radiograph of the head. Both maxilla and mandible are shortened and the lower jaw is malformed with an angulated mandibular body and an elongated mandibular ramus (arrowhead). **(c)** Laterolateral radiograph of the lumbar spine. The vertebral bodies are short, the end plates are poorly mineralized and the dorsal borders of the vertebral spinous processes are irregular. Mediolateral radiograph of **(d)** the right front and **(e)** right hind limbs. The long bones are hourglass-shaped (open arrowheads) and have hypoplastic marrow cavity, as well as hypoplastic cortical bone. Carpal bones and distal femoral epiphyses show irregular mineralization (arrowheads).

Our research group was approached by a KBD breeder concerning a 10-week-old puppy from a litter of nine (litter 1) that had started to fall behind in growth at the age of 7 weeks and now had marked difficulties in walking (Supplementary Video 1). Two littermates of the 10-week-old affected dog had already died at the ages of 3 and 9 weeks without being clinically examined. However, the puppy that had died at 9 weeks of age was reported to have shown similar movement difficulties as the living affected dog. The living puppy was referred for clinical examination at the age of 12 weeks. On admission, the puppy presented with a crouched stance and reluctance to move (Fig. 1a), as well as a generalized muscle weakness, deformed elbow joints on palpation and hyperextension of distal joints. The cognition of the puppy appeared unaffected. Proprioceptive reactions were normal and flexor reflexes fairly strong, although the latter needed a strong stimulus. Euthanasia was elected due to the severity of the clinical signs. Radiographs of spine, skull and limbs were obtained after euthanasia, revealing severe skeletal abnormalities **(**Fig. 1b–e**)**. The most notable changes included thinned, hourglass-shaped diaphyses and poorly mineralized epiphyses and carpal and tarsal bones (Fig. 1d,e).

During the course of the study, we were notified of two additional KBD litters with altogether five affected puppies (four in litter 2 and one in litter 3) that failed to grow similarly to their littermates. At 2 weeks of age, their mean body weight was 646 g (range: 507–902 g), when the average weight of their unaffected littermates was around 1200 g. However, these five puppies were younger than the initially recognized affected dog, and they all had started showing seizures at the age of 2 weeks (Supplementary Video 2). Serum biochemistry profiles were obtained from one affected 2-week-old male puppy and its unaffected male littermate (litter 2) (Supplementary Table 1). The serum analysis revealed elevated total serum protein, albumin and urea levels in the affected dog, which were likely due to clinical dehydration. In addition, when compared to the unaffected sibling, the affected puppy showed a notably decreased serum ALP activity (affected = 10 U/l; control = 98 U/l; adult ref. 20–150 U/l) and an elevated calcium level (affected = 3,09 mmol/l; control = 2,86 mmol/l; adult ref. 2.15–2.95 mmol/l). The affected puppies were euthanized due to their poor condition and referred to a pathological examination at the ages of 14 and 20 days (litter 2 and 3, respectively). Radiographic examination was performed after euthanasia for the four affected puppies in litter 2. The radiographic findings were mostly unremarkable when compared to those of the 12-week-old puppy from litter 1. However, defective mineralization was noted in carpal and small tarsal bones, as well as in epiphyseal areas (Supplementary Fig. 1). Consistent with autosomal recessive mode of inheritance, both sexes were affected. The parents of affected dogs were not clinically examined, but their owners did not report of any related health problems.

### Pathological examinations reveal a mineralization and ossification defect

**Figure 2:**
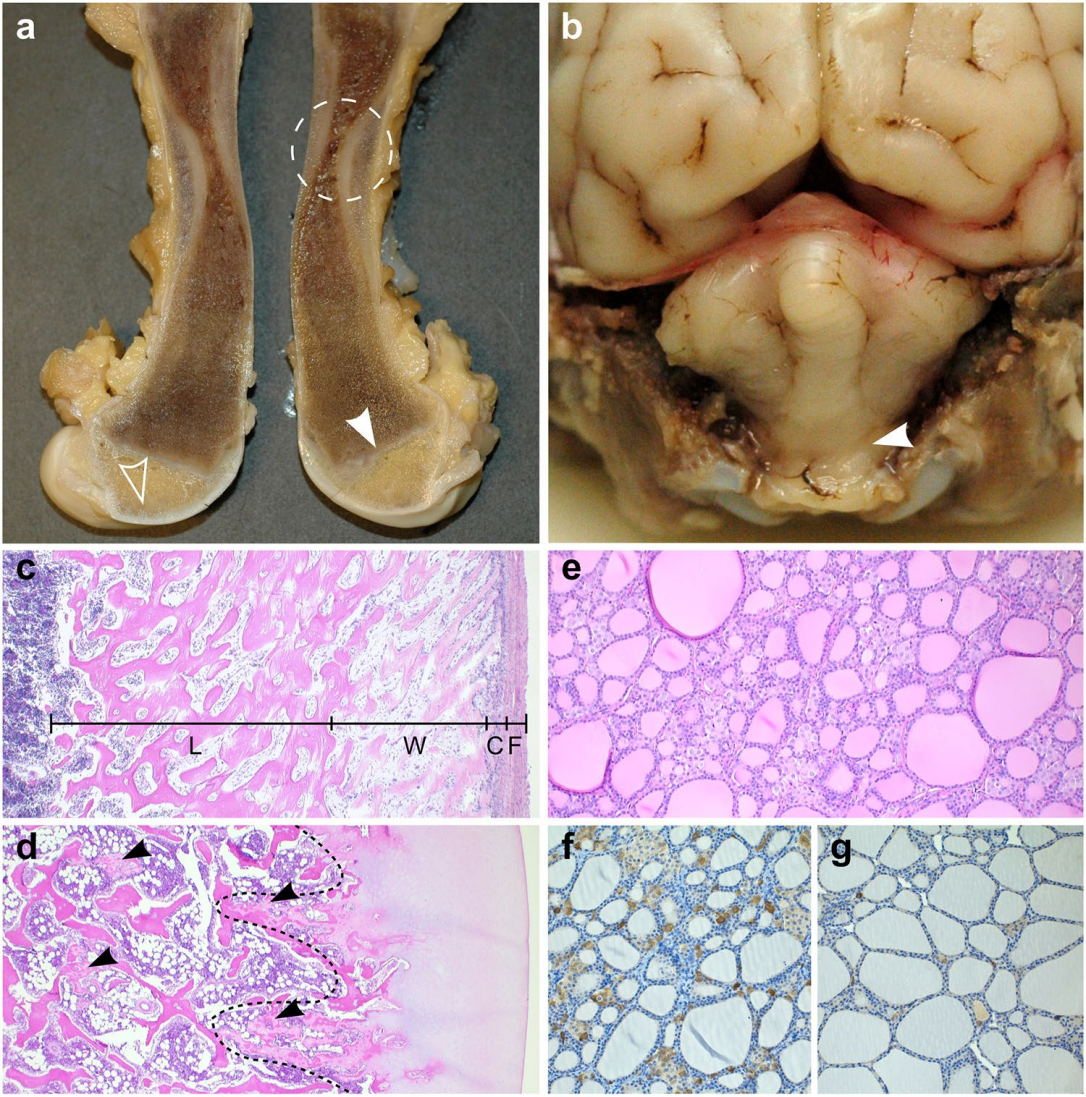
Pathological findings in canine hypophosphatasia. **(a)** A cross section of a formalin fixed femur from a 3-month-old affected puppy (litter 1). The femur has a striking hourglass appearance. The diaphyseal periosteum and cortex are abnormally broadened by unmineralized osteoid and fibrous tissue, and the marrow cavity is narrowed due to defective modelling of primary spongiosa (encircled). Retained epiphyseal cartilage (open arrowhead) and irregular growth lines (arrowhead) are seen within the epiphyseal areas. **(b)** Formalin-fixed cerebellum of a 2-week-old affected puppy (litter 2). The cerebellar vermis is protruding into the spinal canal (arrowhead). **(c)** Longitudinal section of diaphyseal cortex, decalcified, HE 50X. The periosteal cambium (C) is thickened and cellular, with an underlying broad zone of unmineralized woven bone (W) and a cortex consisting of lamellar bone (L) instead of compact bone. The periosteal fibrous layer (F) appears normal. **(d)** Articular cartilage and epiphysis, decalcified, HE 50X. The ossification front is uneven (line) and irregular tongues of retained poorly mineralized cartilage (arrowheads) are present within the epiphysis. **(e)** Thyroid gland from an affected puppy, HE 200X. The C-cells are numerous and hypertrophic between the colloid follicles. **(f)** Thyroid gland from an affected puppy, calcitonin IHC 200X. Profound C-cell hypertrophy and hyperplasia is noted. **(g)** Thyroid gland of an unaffected, age- and gender matched puppy, calcitonin IHC 200X. A few C-cell groups and scattered single calcitonin-rich C-cells are seen. All histopathological images **(c**–**g)** are from the 3-month-old affected puppy (litter 1).

Upon macroscopic examination, the oldest puppy from litter 1 showed marked skeletal changes, namely diaphyseal hypoplasia of long bones (Fig. 2a), which corresponded to the hourglass-like appearance seen in radiological examination. The metaphyses and epiphyses of long bones also showed macroscopic abnormalities, such as persisting primary spongiosa within the metaphyses (Fig. 2a). The skeletal changes were milder in the affected puppies from litters 2 and 3 but some macroscopic findings were noted. Similar to the older affected dog, deficient remodelling of primary spongiosa was detected. In addition, three affected puppies from litter 2 showed cerebellar herniation into foremen magnum (Fig. 2b), a consequence of occipital bone hypoplasia and raised intracranial pressure.

Histologic examination of skeletal tissue revealed similar changes in all affected dogs. Within the diaphyseal and epiphyseal areas of long bones, abnormal bone tissue structure was evident (Fig. 2c,d). Interestingly, the thyroid gland of affected dogs was hypertrophic due to hyperplasia of interfollicular, calcitonin-producing C cells (Fig. 2e–g), a change that occurs as a result of prolonged hypercalcemia^31^. This finding was present in all affected dogs but most prominent in the oldest affected puppy from litter 1. Furthermore, puppies from litter 2 and 3 had mild cerebral oedema, as well as acute lesions related to the cerebellar coning, such as cortical neuronal necrosis in the caudoventral cerebellar vermis. Macroscopic and histologic examination of teeth did not reveal specific findings in the affected dogs. Overall, the pathological findings in affected dogs were compatible with a generalized skeletal ossification and mineralization defect. The specific finding of C cell hyperplasia was indicative of long-term hypercalcemia and compatible with the elevated serum calcium level measured in one affected puppy.

### Exome sequencing reveals a candidate variant in the *ALPL* gene

To identify the genetic cause of the disease, we performed whole exome sequencing for the 3-month-old affected puppy from litter 1. The exome variant data from the affected dog was filtered against 242 control exomes and 658 genomes (Supplementary Table 2), resulting in 346 homozygous case-specific variants. From these variants, we prioritized 25, including all that were predicted to have a protein level impact. Another 10 variants were omitted as they were found in homozygous state in sequence variant data from 13 unaffected KBDs. The remaining 15 variants (Supplementary Table 3) were reviewed in more detail, revealing one highly promising causative candidate, a missense change, c.1301T > G; p.V434G, in the *ALPL* gene (XM_005617214.3, XP_005617271.1).

**Figure 3:**
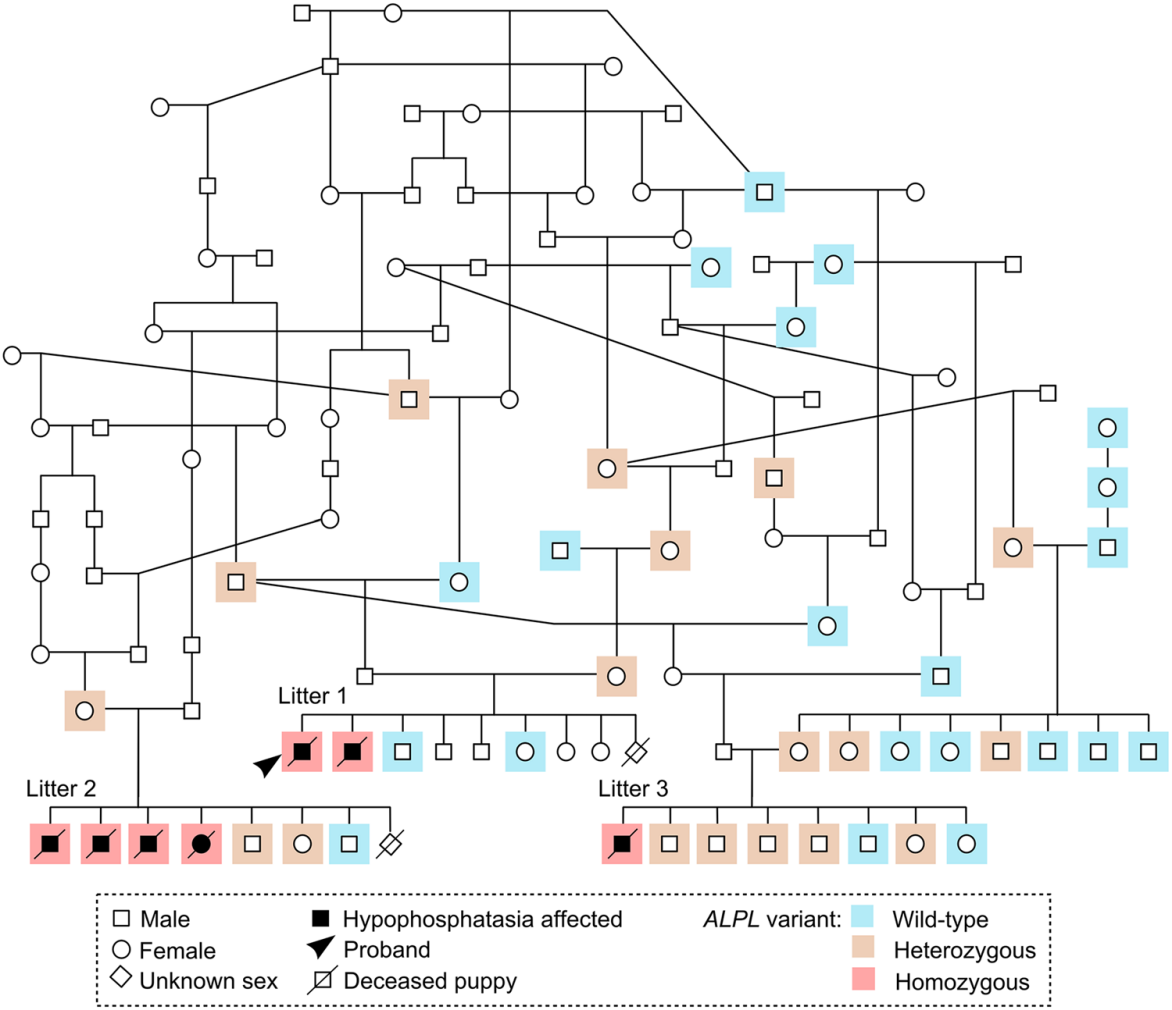
A disease pedigree constructed around the affected dogs. The *ALPL* genotypes are denoted with different colours. Several inbreeding loops and common ancestors are present. Within the affected litters, samples were obtained from altogether three parents and twelve unaffected full siblings. In both litters 1 and 2, one puppy had died without sampling or clinical and pathological examinations, at the age of 3 weeks (litter 1) and 2 days (litter 2). The genotype and possible affection status of these puppies could not therefore be determined.

We proceeded to validate the association between the *ALPL* variant and disease in larger cohorts. DNA samples were obtained from altogether seven affected KBDs, comprising the 3-month-old proband, its sibling that had died at 9 weeks of age, and the five younger puppies from litters 2 and 3. Sanger sequencing of the *ALPL* variant revealed all seven puppies to be homozygous for the missense change (Fig. 3). Furthermore, all dogs from a cohort 281 control KBDs were either heterozygous or wild-type dogs. We then used a Taqman assay to genotype an additional set of 221 unaffected KBDs and a cohort of 303 control dogs from eight different Nordic breeds. The second KBD set did not contain any new variant homozygotes, and all dogs from the other breeds had the reference genotype (Table 1). Overall, these results indicated a full segregation between the *ALPL* variant and the disease. Finally, in order to rule out potential confounding effects on the phenotype, we screened the affected puppies for the previously reported *ITGA10* mutation that causes chondrodysplasia in the breed^29^. The mutation allele was not found in the affected dogs.

**Table 1:** *ALPL* c.1301T > G genotypes in breed cohorts.

Breed	N	T/T	T/G	G/G
Karelian Bear Dog	509	417 (82%)	85 (17%)	7 (1%)
Norwegian Elkhound	88	88	—	—
Finnish Spitz	56	56	—	—
Norrbottenspets	42	42	—	—
Jämthund	40	40	—	—
Finnish Lapphund	31	31	—	—
East Siberian Laika	20	20	—	—
West Siberian Laika	13	13	—	—
Russo-European Laika	13	13	—	—
Total	812	720	85	7

### The *ALPL* variant is located on a conserved protein domain

**Figure 4:**
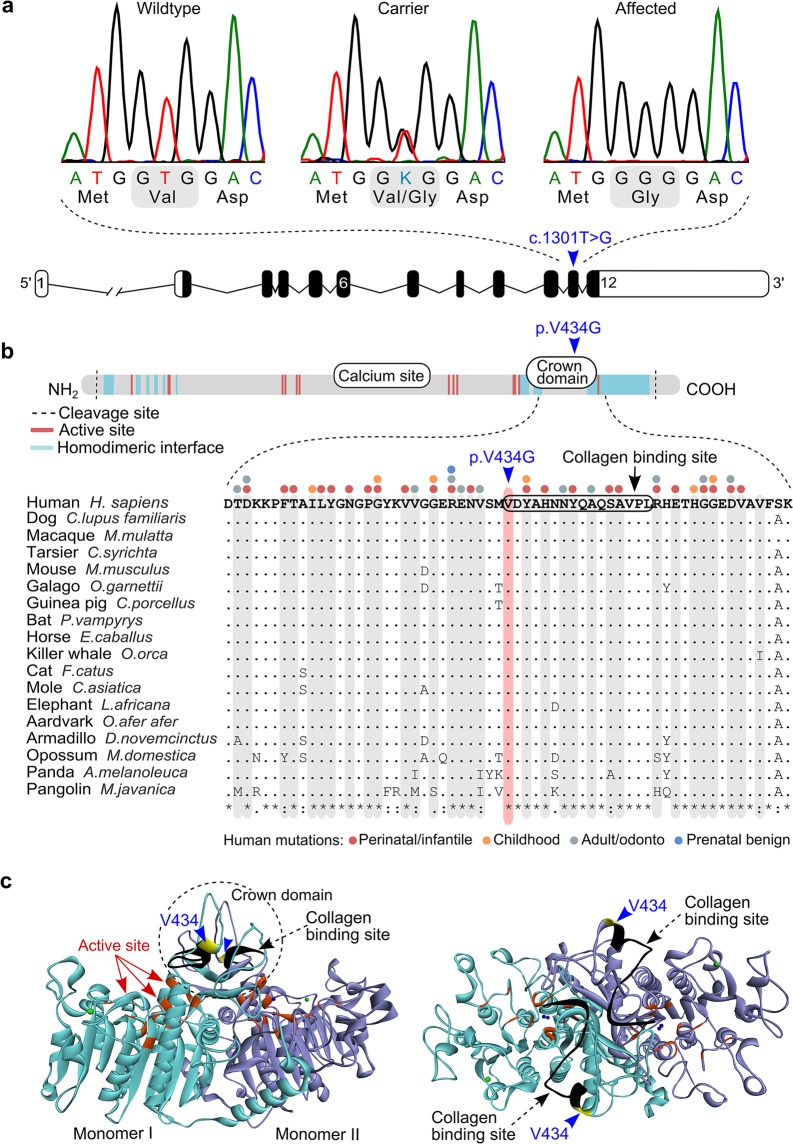
A missense change in the canine *ALPL* gene. **(a)** Sequence chromatograms of the *ALPL* c.1301T > G variant in exon 11 and a schematic representation of the *ALPL* gene. **(b)** Structure of the TNSALP polypeptide and multiple sequence alignment of the variant position in 18 mammalian species. Amino acid residues that make up the active site and the homodimerization interface are scattered along the polypeptide chain, whereas the calcium binding site and the crown domain form separate entities^34^. The p. V434G missense change is located within the crown domain. Those amino acid residues that contain HPP-associated variants in humans are marked on top of the alignment and highlighted in grey (http://www.sesep.uvsq.fr/03_hypo_mutations.php). **(c)** Side and top views of the three-dimensional (3D) structure of the TNSALP homodimer, modelled from the crystal structure of the human placental alkaline phosphatase^32^. The two monomers are separated by different colours. The Val^434^ position is denoted with yellow, the active site residues with red and the collagen binding motif with black. The crown domain forms a flexible loop structure on top of the homodimer.

The identified c.1301T > G change was located on exon 11 of the canine *ALPL* gene, resulting in a valine to glycine amino acid change, p.V434G, in the encoded TNSALP protein (Fig. 4a). In both humans and dogs, the *ALPL* gene is composed of 12 exons, eleven of which are protein coding. The full length TNSALP protein shows 90% sequence identity between the two species, and differs in length by one amino acid, 524 residues in humans (NP_000469.3) and 525 in dogs (XP_005617271.1). The TNSALP polypeptide folds into a cell surface homodimer that contains several known domains, including an active site, homodimeric interface, a calcium-binding site and a crown domain^32–34^. The homozygous p.V434G change in affected dogs was found to be located within the crown domain, more specifically, on the first residue of a reported 16 amino acid long collagen binding site^34^ (Fig. 4b,c). We utilized the *ALPL* Gene Mutation Database (http://www.sesep.uvsq.fr/03_hypo_mutations.php) and the Genome Aggregation Database (gnomAD)^35^ to determine whether any variants have been identified in the Val^434^ position in humans. No such variants were found, but multiple disease-causing mutations were reported in the surrounding sequence (Fig. 4b).

The evolutionary conservation of the Val^434^ was assessed through protein sequence alignments, revealing the Val^434^ position to be conserved in mammals, as well as in most other vertebrate species (Fig. 4b, Supplementary Fig. 2). In-silico pathogenicity predictions tools yielded both neutral and deleterious predictions for the p.V434G change (Supplementary Table 4). The PredictSNP tool^36^, which calculates a consensus estimate from several different algorithms, produced a non-pathogenic prediction (with four neutral and three deleterious predictions). The PROVEAN algorithm^37^ also produced a neutral prediction, whereas the MutationTaster2 algorithm^38^ predicted the p.V434G variant to be disease causing with a very high probability.

### Urine analysis reveals elevated phosphoethanolamine (PEA) levels

In humans, the urine excretion of alkaline phosphatase substrate phosphoethanolamine (PEA) is used as a supporting tool in diagnosing HPP^3,39^. We collected urine samples from three affected dogs during necropsy, from the oldest affected dog in litter 1 and two younger puppies in litter 2. Urine PEA levels were measured from the three affected dogs and from four age-matched control dogs euthanized for other reasons. In support of our genetic findings, all affected KBD puppies excreted markedly elevated levels of PEA ($\dot{{\rm{x}}}$ 579 mmol/mol Crea, range: 300–760) in comparison to the control dogs ($\dot{{\rm{x}}}$ 6,02 mmol/mol Crea, range: 1.46–15.6).

## Discussion

In the present study, we recognize a novel genetic skeletal disease in dogs and identify a homozygous missense change in the *ALPL* gene. *ALPL* mutations cause HPP in humans and the clinical and pathological findings in the affected dogs are compatible with the human disease, particularly the infantile form. Besides the clinicopathological similarities, strong genetic and biochemical evidence of canine HPP was obtained: a fully segregating breed-specific recessive missense variant in a conserved protein domain, combined with serum and urine findings typical for HPP patients.

In humans, most of the known *ALPL* mutations are missense changes (73%) (http://www.sesep.uvsq.fr/03_hypo_mutations.php), which was also the case with the identified canine variant (c.1301T > G; p.V434G). Pathogenicity predictions did not yield a conclusive estimate for the functional impact of the variant, which is likely in part due to the conservative nature of the Val to Gly amino acid change. In human HPP patients, several conservative missense changes (e.g. p. A40V, p.G326V and p.A377V) have been reported across the TNSALP protein (http://www.sesep.uvsq.fr/03_hypo_mutations.php). This suggests that the protein may be particularly vulnerable even to milder amino acid changes, although, the location of the change does play a role. The canine p.V434G variant was positioned within the crown domain of the encoded TNSALP protein. The 65 residues long crown domain is considered essential for protein function, influencing several properties such as enzyme stability and collagen-binding^32,34,40,41^. Our sequence alignments and previous evolutionary analysis^34^ indicate that the Val^434^ position is conserved in mammals and other tetrapods, suggesting that the residue possesses functional significance in higher vertebrates. Causative variants have not been reported in the Val^434^ position in humans, but several disease-causing mutations affect other residues of the crown domain^34^. The Val^434^ residue marks the start of a 16 amino acid long collagen-binding site^34^, and whether the canine variant might affect the collagen binding properties of the TNSALP homodimer, can only be speculated at this point. In humans, several missense variants associated with severe HPP have been shown to result in degradation of the mutant protein by the proteasome system^42^.

The biochemical hallmark of HPP is reduced level of circulating ALP activity, which is inversely correlated with disease severity^43,44^. In humans, ALP activity is higher in children than in adults^45^, which is also true for dogs^46,47^. However, unlike in humans, age-dependent normal ranges are not available for routine biochemistry tests in dogs. We therefore utilized an unaffected sex-matched littermate to help interpret the blood biochemistry results, which revealed the serum ALP level to be nearly 10x lower in the affected 2-week-old puppy than in the healthy sibling. In HPP, the compromised ability of TNSALP to dephosphorylate its natural substrates results in their extracellular accumulation. Increased levels of TNSALP substrates, inorganic pyrophosphate (PPi), pyridoxal 5′-phosphate (PLP) and PEA, has been documented in HPP patients^39,48–50^. In accordance with this, urine analysis uncovered markedly elevated PEA excretion in affected dogs. Although, in terms of HPP pathophysiology, PPi accumulation is considered to be the major pathological mechanism. During bone growth and development, TNSALP is present in osteoblasts and chondrocytes, as well as in osteoblast- and chondrocyte-derived matrix vesicles that serve as the initial site through which mineral is formed and propagated into the collagenous extracellular matrix. The excess amount of unhydrolyzed PPi is thought to inhibit the process of embedding hydroxyapatite (calcium phosphate) crystals into the extracellular matrix, resulting in defective skeletal mineralization^11,51,52^. A corresponding mechanism of teeth mineralization, involving MVs budding from odontoblasts, explains the dental phenotype in HPP patients^53,54^.

In humans, the *ALPL* genotype of HPP patients has been shown to correlate fairly well with the clinical phenotype^55,56^. However, variable expressivity has been reported in some patients with identical genotypes, and whether this variation is caused by genetic or epigenetic modifiers or other confounding factors is not known^57^. Phenotypic variation was also present in affected dogs, in which two clinical presentations were recognized. The earlier onset disease progressed to owner-elected euthanasia before 3 weeks of age due to seizures and failure to thrive (litters 2 and 3), whereas the other form became apparent after one month of age and developed into a generalized ossification defect (litter 1). In humans, seizures have been described in perinatal and infantile HPP and they serve as a strong indicator for a lethal outcome^58^. The underlying cause of the neonatal seizures is not well understood. However, in several cases, the seizures have been responsive to pyridoxine (vitamin B6) treatment rather than to standard antiepileptic drugs^59–63^, which may be explained by the role of TNSALP in vitamin B6 metabolism. Extracellular PLP (the active form of vitamin B6) is dephosphorylated by TNSALP, enabling its uptake into cells as pyridoxal and subsequent rephosphorylation back to PLP^64^. Intracellular vitamin B6 functions as coenzyme in neurotransmitter metabolism, and therefore, low pyridoxal levels have been suggested to cause seizures through a defect in neurotransmitter synthesis^8,60,65^. In the affected KBDs, treatment of the seizures was not attempted.

Radiologic and histopathologic examinations of affected dogs revealed a skeletal mineralization and ossification defect. Dental abnormalities were not detected. The skeletal changes were more severe in the oldest affected dog, including pronounced diaphyseal hypoplasia and generalized hypomineralization. The younger puppies had milder skeletal changes, however, in three of them, cerebellar herniation was noted. In human infantile and childhood HPP, premature closure of the cranial sutures (craniosynostosis) is a common manifestation, which can lead to increased intracranial pressure and other complications, such as herniation of the cerebellar tonsils^66^. Another finding in the affected KBDs was hyperplasia of the thyroidal C cells. The C cells of the thyroid gland release the hormone calcitonin in response to elevated blood calcium levels, and in the case of prolonged hypercalcemia, C cell hyperplasia occurs^31^. This histological finding was supported by the increased serum calcium levels measured in one affected dog. Hypercalcemia is reported in human HPP patients as well, resulting from decreased entry of minerals into the skeleton. In extreme cases, this can lead to nephrocalcinosis and renal failure^17^. In the affected dogs, metastatic calcification of soft tissues was not detected.

In conclusion, our study reports a strong association between a skeletal mineralization defect in dogs and a recessive variant in a conserved domain of the *ALPL* gene. Together, our clinical, pathological and genetic findings strongly suggest that the skeletal phenotype observed in affected dogs is HPP, resembling the infantile disease form in humans. Our findings represent the first report of naturally occurring HPP in animals and have enabled the development of a genetic test for breeding purposes.

## Materials and Methods

### Ethical approval

All dogs included in this study were privately owned pets. Sample collections, clinical examinations and animal necropsies were carried out with the dog owners’ informed consent. The experiments were approved by the Finnish national Animal Experiment Board (ESAVI/7482/04.10.07/2015) and performed in accordance with relevant guidelines and regulations.

### Serum and urine analysis

Serum biochemistry profiles (albumin, alkaline phosphatase, alanine aminotransferase, amylase, total bilirubin, blood urea nitrogen, calcium, phosphorus, creatinine, glucose, sodium, potassium, total protein and globulin) were examined from one affected and one control puppy (Supplementary Table 1). Urine samples were obtained from three affected dogs (one puppy from litter 1 and two from litter 2) and three age-matched control dogs, euthanized due to cerebellar dysplasia (n = 2) and congenital hypothyroidism (n = 1). The samples were collected at autopsy via direct open cystocentesis. The amount of excreted phosphoethanolamine (PEA) was analysed from the urine samples by high performance liquid chromatography (Synlab) and the urinary concentration differences were normalized against the amount of creatine.

### Pathological examinations

One puppy from litter 1 underwent post-mortem examination at the Companion Animal Pathology Section in the Finnish Food Safety Authority Evira and six puppies were necropsied at the Section of Veterinary Pathology, University of Helsinki. The puppy necropsied at Evira had died naturally, whereas the other affected dogs were euthanized on their owners’ request. The post mortem examinations included gross examination of the body and organs, including sectioning of long bones and spine in selected cases. Histologic examination of brain and internal organs was performed for all puppies. Specific histological sampling of skeletal, dental and muscular tissue was carried out for six dogs after the candidate variant was identified. Fresh sampled tissues were formalin fixed, paraffin-embedded and routinely stained with hematoxylin-eosin (HE). To verify C-cell hyperplasia, thyroid glands were immunohistochemically stained with an anti-calcitonin antibody (ab 75368, prediluted Abcam).

### Study cohorts and DNA samples

Canine samples were retrieved from the dog DNA bank at the University of Helsinki. The KBD cohort comprised altogether 509 dogs, including seven affected dogs (Table 1). The sample of one affected KBD puppy was received from Finnish Food Safety Authority Evira, where the animal had been necropsied. A control cohort of Nordic breeds comprised 303 dogs from eight different breeds (Table 1). Genomic DNA was isolated from EDTA blood or tissue (in the case of a few necropsied dogs) by using a semi-automated DNA extraction robot (PerkinElmer chemagen Technologie GmbH). DNA concentrations were measured by using either a Nanodrop ND-1000 UV/Vis Spectrophotometer (Nanodrop technologies) or a Qubit fluorometer (Thermo Fisher Scientific).

### Pedigrees

Finnish Kennel Club’s pedigree registry KoiraNet (http://jalostus.kennelliitto.fi/) was utilized to retrieve pedigree information for the affected dogs and the GenoPro genealogy software (http://www.genopro.com/) was used to draw the disease pedigree.

### Reference sequences

The dog genome build CanFam 3.1 was used in all analyses. The canine *ALPL* mRNA and protein reference sequences were XM_005617214.3 and XP_005617271.1, respectively.

### Next generation sequencing

Whole exome sequencing of an affected dog was performed by using Roche NimbleGen SeqCap EZ target enrichment design 140702_canFam3_exomeplus_BB_EZ_HX1 with a capture size of ~152 Mb^67^. The sequencing was carried out at the Biomedicum Functional Genomics Unit (FuGU, University of Helsinki) using Illumina NextSeq500 technology. The Burrows-Wheeler Aligner (BWA) version 0.7.12-r1039^68^ was used to map the acquired reads and the Picard tools (http://broadinstitute.github.io/picard/) to sort the mapped reads and to mark duplicates. Indel realignment, base-quality score recalibration and variant calling was carried out using the Genome Analysis Tool Kit (GATK) HaplotypeCaller, version 3.5.0^69^. The sequencing yielded ~83 million 2 × 150 bp paired-end reads, of which 99,5% were mapped to the dog genome (CanFam 3.1) with a 60X mean coverage. Variant calling produced 883,008 single nucleotide variants and 292,075 small insertions and deletions. To identify potential causative variants in the affected dog, we performed variant filtering under the assumption of autosomal recessive inheritance and a breed-specific founder mutation. For this purpose, we used our in-house variant database, which was queried using Genotype Query Tools^70^. The entire sequence data cohort utilized in this study comprised altogether 252 whole exomes and 663 whole genomes from 903 animals, including the exome of an affected KBD, as well as nine exomes and five genomes from 13 unaffected KBDs (Supplementary Table 2). However, the unaffected KBDs were omitted from the initial variant filtering step in case they were carriers of the causative variant. The dog and wolf genome SNP database (DoGSD)^71^ was utilized to retrieve 38 control genomes.

### Variant validation

Sanger sequencing and Taqman genotyping were utilized to screen the *ALPL* variant in our study samples. Sanger sequencing primers, GTTCTCCAACCTGACTCCTG and AGAGAGGCAGAGTTCGCATG (5′ > 3′ forward and reverse, respectively), were designed with the Primer3 program (http://bioinfo.ut.ee/primer3/)^72^. PCR amplification was carried out by using Biotools DNA Polymerase (Biotools B&M Labs, S.A.) and sequencing reactions were performed at the Institute for Molecular Medicine Finland (FIMM). The Sequencher (version 5.2.4) program (Gene Codes Corporation) was used to analyse the resulting Sanger sequence data. A custom Taqman SNP genotyping assay was utilized to genotype additional sample cohorts (ThermoFisher Scientific). The primer and probe sequences for the assay were the following: CGGTGAGCGAGAGAACGT and CCCAGCCCCGGATTTGG (5′ > 3′ forward and reverse, respectively), VIC-CCTCACCGTAGTCCACCAT and FAM-CTCACCGTAGTCCCCCAT (5′ > 3′ wild-type and variant allele, respectively). The Taqman genotyping reactions were carried out using Taqman Genotyping Master Mix (ThermoFisher Scientific) and Biorad’s CFX96 Touch Real-Time PCR Detection System. The *ITGA10* variant was genotyped from the affected dogs by using Sanger sequencing as described previously^29^.

### Bioinformatic analyses of the *ALPL* variant

To visualize the native structure of the TNSALP homodimer, a three-dimensional (3D) model of the protein was retrieved from the SWISS-MODEL Repository (https://swissmodel.expasy.org/). We utilized a model of the human TNSALP (UniProtKB accession: P05186) that had been constructed on the basis of the crystal structure of the human placental alkaline phosphatase (template ID: 1zef.1 A)^32^. The 3D image was drawn by using the Discovery Studio 4.5 Visualizer (BIOVIA). Multiple sequence alignments of the TNSALP protein were constructed by using the Clustal Omega algorithm (https://www.ebi.ac.uk/Tools/msa/clustalo/)^73^. The aligned protein sequences were retrieved from the Entrez protein database (https://www.ncbi.nlm.nih.gov/protein/) and their respective sequence identifiers are found in Supplementary Fig. 2. The pathogenicity of the *ALPL* variant was assessed through the PROVEAN (http://provean.jcvi.org/index.php)^37^, PredictSNP (https://loschmidt.chemi.muni.cz/predictsnp1/)^36^ and MutationTaster2 (http://www.mutationtaster.org/)^38^ algorithms. The first two programs were used with the canine *ALPL* reference sequence (XM_005617214.3, XP_005617271.1), whereas the latter was used with a human reference (NM_000478.5, NP_000469.3).

## Supplementary information

Dataset 1

Supplementary Video 1

Supplementary Video 2

## Data Availability

The exome sequence data of the affected dog has been submitted to the Sequence Read Archive (SRA) (https://www.ncbi.nlm.nih.gov/sra) and will be available with a study accession SRP142577.
