# Supplementary material for: A homozygous missense variant in the alkaline phosphatase gene ALPL is associated with a severe form of canine hypophosphatasia
Source: Sci Rep. 2019 Jan 30;9:973. doi: 10.1038/s41598-018-37801-2 (PMC6353930; doi:10.1038/s41598-018-37801-2)
Supplement: Supplementary file 1 — Dataset 1 [file 41598_2018_37801_MOESM1_ESM.pdf]

SUPPLEMENTARY MATERIAL

A homozygous missense variant in the alkaline phosphatase gene *ALPL* is associated with a severe form of canine hypophosphatasia

Kaisa Kyöstilä[1,2,3], Pernilla Syrjä[1], Anu K. Lappalainen[4], Meharji Arumilli[1,2,3], Sruthi Hundi[1,2,3], Veera Karkamo[5], Ranno Viitmaa[4], Marjo K. Hytönen[1,2,3], Hannes Lohi[1,2,3*]

[1] Department of Veterinary Biosciences, University of Helsinki, 00014 Helsinki, Finland

[2] Research Programs Unit, Molecular Neurology, University of Helsinki, 00014 Helsinki, Finland

[3] Department of Molecular Genetics, Folkhälsan Institute of Genetics, 00290 Helsinki, Finland

[4] Department of Equine and Small Animal Medicine, University of Helsinki, 00014 Helsinki, Finland

[5] Veterinary Bacteriology and Pathology Research Unit, Finnish Food Safety Authority Evira, 00790 Helsinki, Finland.

*Corresponding author: Hannes Lohi, PhD, Professor
Biomedicum Helsinki, Haartmaninkatu 8, P.O.Box 63
University of Helsinki, 00014 Helsinki, Finland
E-mail: hannes.lohi@helsinki.fi, Tel: +358 2 941 2508

CONTENTS

Supplementary Figure 1. Radiographic findings in 2-week-old affected dogs.

Supplementary Figure 2. Conservation of the Val^{434} position in 136 vertebrate species.

Supplementary Table 1. Serum biochemistry in an affected and healthy 2-week-old littermate.

Supplementary Table 2. Samples used in NGS variant filtering.

Supplementary Table 3. Prioritized homozygous variants in the affected dog.

Supplementary Table 4. Pathogenicity prediction results.

Supplementary Video Legends:

- **Supplementary Video 1.** An affected puppy at 10 weeks of age (litter 1).
- **Supplementary Video 2.** An affected puppy at 2 weeks of age (litter 2).

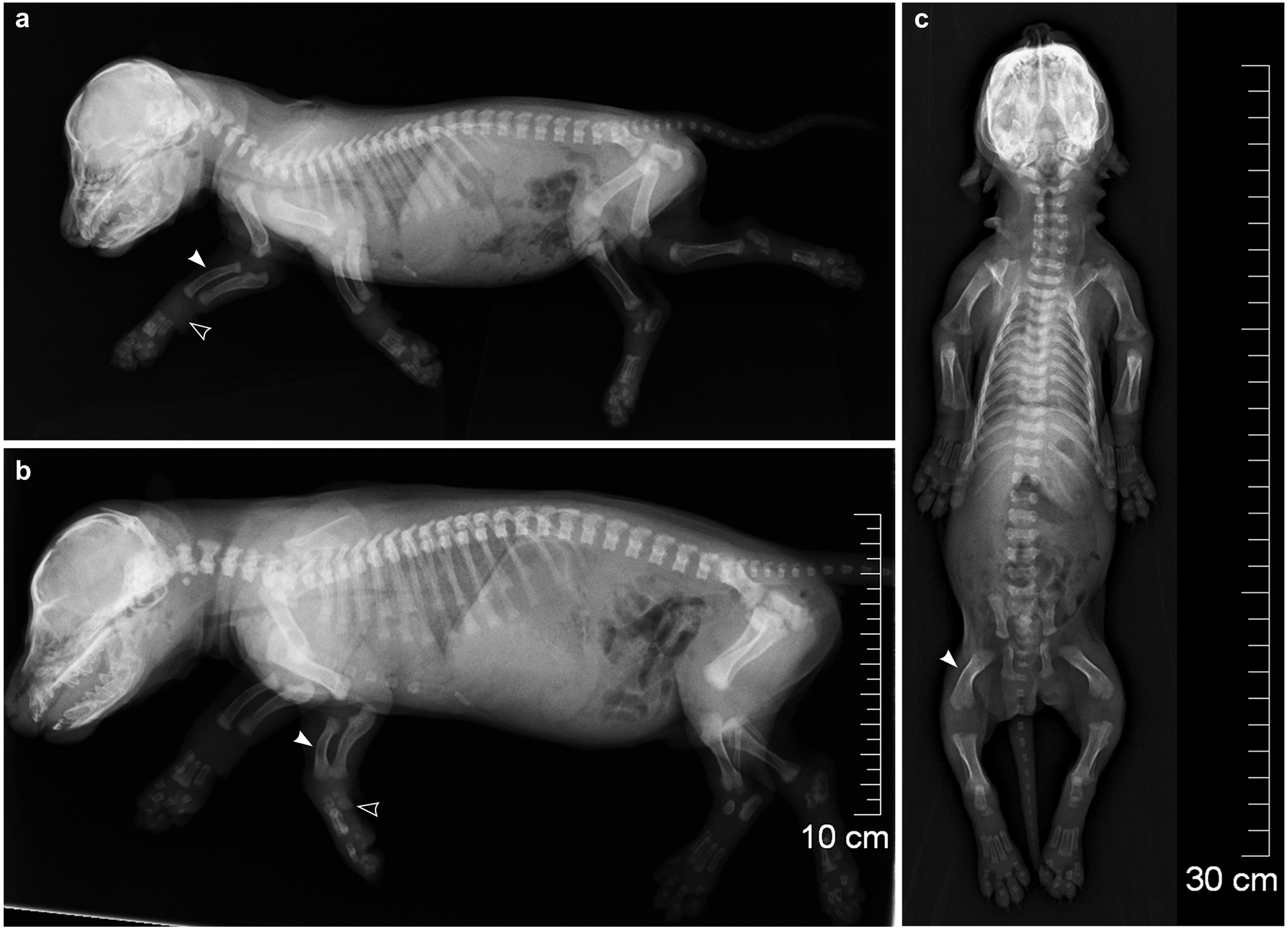

Supplementary Figure 1. **Radiographic findings in 2-week-old affected dogs. (a)** Carpal bones are non-mineralized (open arrowhead) but radius appears normal (arrowhead). **(b)** Mineralized carpal bones in one limb (open arrowhead). Radius is bowed cranially (arrowhead). **(c)** Right femur is abnormally curved (arrowhead).

**FISH** *C.milii* XP_007896010.1	DIDHKPFTSILYGNGPGYKLASGERRNLSSVDINAKSYLAQSAVPLKQETHGGEDVAIFAK
**FISH** *L.chalumnae* XP_005988748.1	DVDNKPFTSILYGNGPGYKVINGERENVSNIDIHYNNYLAQSAVPLRQETHGGEDVAVFAK
**FISH** *L.oculatus* XP_006641996.1	DIDQKPFTSILYGNGPGYKLVNGGRENVSTTDYNHNDYLAQSAVPLRMETHGGEDVAIFSK
**FISH** *S.grahami* XP_016109349.1	DIDQKPFTSILYGNGPGFKFVNGARENVSTVDYEHNNYQAQAAVPLSMETHGGEDVAIFSK
**ZEBRAFISH** *D.rerio* XP_005169330.1	DVDQKPFTAILYGNGPGFKLVNGARENVSTVDYQQNNYQAQSAVPLRMETHGGEDVAIFSK
**CATFISH** *I.punctatus* XP_017306457.1	DVDQKPFTSILYGNGPGFKLINGSRVNVSTVDYKENNYQAQAAVPLSMETHGGEDVAIFSK
**PIRANHA** *P.nattereri* XP_017573422.1	DVDQKPFTSILYGNGPGFKLVNGSRVNVSTVDYQQNDYQAQAAVPLRMETHGGEDVAVFAK
**HERRING** *C.harengus* XP_012683429.1	DIDQKPFTSILYGNGPGFKLIDGLRENVSTVDYQQNNYQAQSAVPLRFETHGGEDVAVFSK
**FISH** *N.furzeri* XP_015813883.1	DADQKPFTAISYGNGPGYKIVNGGRENISTVNYESSHYQAQAAVPLLSETHGGDDVAVFAK
**FISH** *O.latipes* XP_004069357.1	DVDHKPFTSILYGNGPGYKLVGGARENVSTVDFAENNYKAQSAVPLTSETHGGEDVAVFAK
**PIKE** *E.lucius* XP_010879964.1	DVDQKPFTAILYGNGPGFKVVDGLRENVSTIDYQENNYQAQAAVPLSMETHGGEDVAIFSK
**FISH** *X.maculatus* XP_014325431.1	DVDHKSYTSILYGNGPGYKLVKGERENASVAKYDEKNYQAQAAVPLSSETHGGEDVAVFAK
**MILLIONFISH** *P.reticulata* XP_008407325.1	DVDHKSYTSILYGNGPGYKLVXGERENASVTKYEEKNYQAQAAVPLSMETHGGEDVAVFAK
**FISH** *P.formosa* XP_007547072.1	DVDHKSYTSILYGNGPGYKLVKGERENASVTKYEEKNYQAQAAVPLSAETHGGEDVAVFAK
**FISH** *P.latipinna* XP_014908094.1	DVDHKSYTSILYGNGPGYKLVKGERENASVTKYEEKNYQAQAAVPLSAETHGGEDVAVFAK
**FISH** *C.semilaevis* XP_008318967.1	DVDQMSFTSILYGNGPGYKVVRGSRENVSTVDYMDNNYQAQSAVPLAMETHGGEDVAVFAK
**PUFFERFISH** *T.rubripes* XP_011611897.1	DVDQKPFTAILYGNGPGYKLVNGGRENVSAIDYQENNYQAQAAVPLSMETHGGEDVAVFAK
**FISH** *F.heteroclitus* XP_012737184.1	DVDNKHFTAISYGNGPGYKLIDGQRENVSKIKSEDNNYQAQAAVPLRTETHGGEDVAVFAK
**FISH** *C.variegatus* XP_015230832.1	DIDQKPFTSILYGNGPGYKLVNGARENVSTIKYEDNNYRAQSAVPLNYETHGGEDVAVFAK
**FISH** *O.niloticus* XP_003447731.1	DVDQKPFTSILYGNGPGYKVINGARENVSTVDYKGNNYQAQAAVPLNAETHGGEDVAVFAK
**FISH** *P.nyererei* XP_005729746.1	DVDQKPFTSILYGNGPGYKVINGARENVSAVDYKGNNYQAQAAVPLNAETHGGEDVAVFAK
**FISH** *H.burtoni* XP_005917932.1	DVDQKPFTSILYGNGPGYKVINGARENVSAVDYKGNNYQAQAAVPLNAETHGGEDVAVFAK
**FISH** *M.zebra* XP_004547515.1	DVDQKPFTSILYGNGPGYKVINGARENVSAVDYKGNNYQAQAAVPLNAETHGGEDVAVFAK
**FISH** *S.partitus* XP_008304484.1	DIDQKPFTSILYGNGPGYKVVNGARENVSAIDYQENNYQAQAAVPLSMETHGGEDVAVFAK
**FISH** *L.crocea* NP_001290269.1	DVDQKPFTSILYGNGPGYKLVNGARENVSTIDYQENNYQAQSAVPLSMETHGGEDVAVFAK
**FROG** *X.laevis* NP_001080895.1	DVDQKPFTSILYGNGPGYKLVNGQRENVSTVDFSHPNYLAQSAVPLRMETHGGEDVAVFAK
**TURTLE** *C.p.bellii* XP_005281771.1	DVDKMPFTSILYGNGPGYKIVAGERENVSVVDYAHANYQAQSAVPLRQETHGGEDVAIFAK
**ALLIGATOR** *A.mississippiensis* XP_006261757.2	DVDKKPFTSILYGNGPGYKLIGGGRENVSTVDFAHANYQAQSAVPLRQETHGGEDVAIFAK
**ALLIGATOR** *A.sinensis* XP_006023251.1	DVDKKPFTSILYGNGPGYKLIGGGRENVSTVNFAHANYQAQSAVPLRQETHGGEDVAVFAK
**GEKKO** *G.japonicus* XP_015268119.1	DIDKKPFTSILYGNGPGYKVVAGERENVSWVDFAHANYQAQSAVPLRQETHGGEDVAVFAK
**SNAKE** *T.sirtalis* XP_013925283.1	DMDKKPFTSILYGNGPGYKIIAGERENISAVNFTDANYQAQSAVPLRMETHGGEDVAVFAK
**PYTHON** *P.bivittatus* XP_007443195.1	DVDKKPFTSILYGNGPGYRMVAGERENISVVNFTDTNYQAQSAVPLRMETHGGEDVAVFAK
**VIPER** *P.mucrosquamatus* XP_015682899.1	DMDKKPFTSILYGNGPGYKMVAGERENVSAVNFTDTNYQAQSAVPLRMETHGGEDVAVFAK
**HUMMINGBIRD** *C.anna* XP_008502009.1	DVDRKPFTSILYGNGPGYKIVAGERENVSAVDFAHADYQAQSAVPLRQETHGGEDVAVFAR
**IBIS** *N.nippon* XP_009461739.1	DVDRKPFTSILYGNGPGYKIVAGERENVSAVDFAHANYQAQSAVPLRQETHGGEDVAVFAR
**BIRD** *M.unicolor* XP_010183946.1	DVDRKPFTSILYGNGPGYKIVAGERENVSAVDFAHADYQAQSAVPLRQETHGGEDVAVFAR
**BIRD** F.albicollis XP_016158991.1	DVDRKPFTSILYGNGPGYKIVAGERENVSAVDFAHADYQAQSAVPLRQETHGGEDVAVFAR
**BIRD** *S.vulgaris* XP_014743896.1|	DVDRKPFTSILYGNGPGYKIVAGERENVSAVDFAHADYQAQSAVPLRQETHGGEDVAVFAR
**GREAT TIT** *P.major* XP_015503571.1	DVDRKPFTSILYGNGPGYKIVAGERENVSAVDFAHADYQAQSAVPLRQETHGGEDVAVFAR
**GROUND TIT** *P.humilis* XP_005533217.1	DVDRKPFTSILYGNGPGYKIVAGERENVSAVDFAHADYQAQSAVPLRQETHGGEDVAVFAR
**CANARY** *S.canaria* XP_009093893.1	DVDRKPFTSILYGNGPGYKIVAGERENVSAVDFAHADYQAQSAVPLRQETHGGEDVAVFAR
**SPARROW** *Z.albicollis* XP_014129865.1	DVDRKPFTSILYGNGPGYKIVAGERENVSAVDFAHADYQAQSAVPLRQETHGGEDVAVFAR
**CHICKEN** *G.gallus* NP_990691.1	DVDRKPFTSILYGNGPGYKIVGGERENVSAVDFAHANYQAQAAVPLRQETHGGEDVAVFAR
**QUAIL** *C.japonica* XP_015737738.1	DVDRKPFTSILYGNGPGYKIVGGERENVSAVDFAHANYQAQAAVPLRQETHGGEDVAVLAR
**CUCKOO** *C.canorus* XP_009562601.1	DVDRKPFTSILYGNGPGYKIVAGERENVSAVDFAHANYQAQSAVPLRQETHGGEDVAVFAR
**EAGLE** *A.c.canadensis* XP_011583331.1	DVDRKPFTSILYGNGPGYKIVAGERENVSAVDFAHANYQAQSAVPLRQETHGGEDVAVFAR
**EAGLE** *H.leucocephalus* XP_010575495.1	DVDRKPFTSILYGNGPGYKIVAGERENVSAVDFAHANYQAQSAVPLRQETHGGEDVAVFAR
**FALCON** *F.peregrinus* XP_005237645.1	DVDRKPFTSILYGNGPGYKIVAGERENVSAVDFAHANYQAQSAVPLRQETHGGEDVAVFAR
**FALCON** *F.cherrug* XP_005437898.1	DVDRKPFTSILYGNGPGYKIVAGERENVSAVDFAHANYQAQSAVPLRQETHGGEDVAVFAR
**OPOSSUM** M.domestica XP_007491291.1	DTDNKPYTSILYGNGPGYKVVAGQRENVSTVDYAHDNYQAQSAVPLSYETHGGEDVAVFAK
**TASMANIAN DEVIL** *S.harrisii* XP_012400932.1	DRDNKPFTSILYGNGPGYRIVAGERENIFTVDYAHENYQAQSAVPLGSETHGGEDVAVFAK
**PANGOLIN** *M.javanica* XP_017506628.1	DMDRKPFTAILYGNGPGFRVMGSERENISVVDYAHKNYQAQSAVPLHQETHGGEDVAVFAK
**SHREW** *S.araneus* XP_012787053.1	DTDKKPFTSILYGNGPGYKVVGGERENVSMVDYAHNNYQAQSAVPLRHETHGGEDVAVFAK
**PANDA** A.melanoleuca XP_002919566.1	DTDNKPFTAILYGNGPGYKVIGGERENIYKVDYAHSNYQAQAAVPLRYETHGGEDVAVFAK
**POLAR BEAR** *U.maritimus* XP_011741978.1	DTDNKPFTAILYGNGPGYKVVGGERENIYTVDYAHSNYQAQSAVPLRYETHGGEDVAVFAK
**PIKA** *O.princeps* XP_004592343.1	DTDKKPFTAILYGNGPGYKVVGGERENVSMVDYAHNNYQAQSAVPLRHETHGGEDVAVFAK
**SEAL** *L.weddellii* XP_006744511.1	DTDKKPFTAILYGNGPGYKVVGGERENVSMVDYAHNNYQAQSAVPLRHETHGGEDVAVFAK
**TREESHREW** *T.chinensis* XP_006145237.1	DTDKKPFTAILYGNGPGYKVVGGERENVSMVDYAHNNYQAQSAVPLRHETHGGEDVAVFAK
**FERRET** *M.p.furo* XP_004741321.1	DTDKKPFTSILYGNGPGYKVVGGERENVSMVDYAHNNYQAQSAVPLRYETHGGEDVAVFAK
**TIGER** *P.t.altaica* XP_007083789.1	DTDKKPFTSILYGNGPGYKVVGGERENVSMVDYAHNNYQAQSAVPLRHETHGGEDVAVFAK
**CHEETAH** *A.jubatus* XP_014941877.1	DTDKKPFTSILYGNGPGYKVVGGERENVSMVDYAHNNYQAQSAVPLRHETHGGEDVAVFAK
**CAT** *F.catus* NP_001036028.1	DTDKKPFTSILYGNGPGYKVVGGERENVSMVDYAHNNYQAQSAVPLRHETHGGEDVAVFAK
**DOG** *C.l.familiaris* XP_005617271.1	DTDKKPFTAILYGNGPGYKVVGGERENVSMVDYAHNNYQAQSAVPLRHETHGGEDVAVFAK
**WALRUS** *O.r.divergens* XP_004411767.1	DTDKKPFTAILYGNGPGYKVVGGERENVSMVDYAHNNYQAQSAVPLRHETHGGEDVAVFAK
**MOLE** *C.cristata* XP_004678632.1	DADKKPFTSILYGNGPGYKVVGGERENVSMVDYAHNNYQAQSAVPLRHETHGGEDVAVFAK
**ANTELOPE** *P.hodgsonii* XP_005957095.1	DTDKKPFTAILYGNGPGYKVVGGERENVSMVDYAHNNYQAQSAVPLRHETHGGEDVAVFAK
**GOAT** *C.hircus* XP_013830058.1	DTDKKPFTAILYGNGPGYKVVGGERENVSMVDYAHNNYQAQSAVPLRHETHGGEDVAVFAK
**WATER BUFFALO** *B.bubalis* XP_006048607.1	DTDKKPFTAILYGNGPGYKVVGGERENVSMVDYAHNNYQAQSAVPLRHETHGGEDVAVFAK
**COW** *B.taurus* XP_015315483.1	DTDKKPFTAILYGNGPGYKVVGGERENVSMVDYAHNNYQAQSAVPLRHETHGGEDVAVFAK
**BISON** B.b.bison XP_010828286.1	DTDKKPFTAILYGNGPGYKVVGGERENVSMVDYAHNNYQAQSAVPLRHETHGGEDVAVFAK
**YAK** *B.mutus* XP_005905265.1	DTDKKPFTAILYGNGPGYKVVGGERENVSMVDYAHNNYQAQSAVPLRHETHGGEDVAVFAK
**BAT** *M.natalensis* XP_016070047.1	DTDKKPFTAILYGNGPGYKVVGGERENVSMVDYAHNNYQAQSAVPLRHETHGGEDVAVFAK
**BAT** *E.f.eptesicus* XP_008146346.1	DTDKKPFTAILYGNGPGYKVVGGERENVSMVDYAHNNYQAQSAVPLRHETHGGEDVAVFAK
**BAT** *P.vampyrus* XP_011356324.1	DTDKKPFTAILYGNGPGYKVVGGERENVSMVDYAHNNYQAQSAVPLRHETHGGEDVAVFAK
**BAT** *P.alecto* XP_006924536.1	DTDKKPFTAILYGNGPGYKVVGGERENVSMVDYAHNNYQAQSAVPLRHETHGGEDVAVFAK
**BAT** *R.aegyptiacus* XP_016004729.1	DTDKKPFTAILYGNGPGYKVVDGERENVSMVDYAHNNYQAQSAVPLRHETHGGEDVAVFAK
**BAT** *M.davidii* XP_006777666.1	DTDKKPFTSILYGNGPGYKVVGGERENVSMVDYAHNNYQAQSAVPLRHETHGGEDVAVFAK

```
BAT M.brandtii XP_005859737.1                     DTDKKPFTSILYGNGPGYKVVGGERENVSMVDYAHNNYQAQSAVPLRHETHGGEDVAVFAK
BAT M.lucifugus XP_006094076.1                    DTDKKPFTSILYGNGPGYKVVGGERENVSMVDYAHNNYQAQSAVPLRHETHGGEDVAVFAK
DONKEY E.asinus XP_014710606.1                    DTDKKPFTAILYGNGPGYKVVGGERENVSMVDYAHNNYQAQSAVPLRHETHGGEDVAVFAK
HORSE E.caballus XP_005607435.1                   DTDKKPFTAILYGNGPGYKVVGGERENVSMVDYAHNNYQAQSAVPLRHETHGGEDVAVFAK
ALPACA V.pacos XP_015094982.1                     DTDKKPFTAILYGNGPGYKVVGGERENVSMVDYAHNNYQAQSAVPLRHETHGGEDVAVFAK
DROMEDARY C.dromedarius XP_010978117.1            DTDKKPFTAILYGNGPGYKVVGGERENVSMVDYAHNNYQAQSAVPLRHETHGGEDVAVFAK
CAMEL C.bactrianus XP_010955716.1                 DTDKKPFTAILYGNGPGYKVVGGERENVSMVDYAHNNYQAQSAVPLRHETHGGEDVAVFAK
CAMEL C.ferus XP_006188361.2                      DTDKKPFTAILYGNGPGYKVVGGERENVSMVDYAHNNYQAQSAVPLRHETHGGEDVAVFAK
PIG S.scrofa XP_020953341.1                       DTDKKPFTAILYGNGPGYKVVGGERENVSMVDYAHDNYQAQSAVPLRHETHGGEDVAIFAR
WHALE B.a.scammoni XP_007175134.1                 DTDKKPFTAILYGNGPGYKVVGGERENVSMVDYAHDNYQAQSAVPLRHETHGGEDVAVFAK
DOLPHIN T.truncatus XP_004318166.1                DTDKKPFTAILYGNGPGYKVVGGERENVSMVDYAHNNYQAQSAVPLRHETHGGEDVAIFAK
KILLER WHALE O.orca XP_004272548.1                DTDKKPFTAILYGNGPGYKVVGGERENVSMVDYAHNNYQAQSAVPLRHETHGGEDVAIFAK
DOLPHIN L.vexillifer XP_007459278.1               DTDKKPFTAILYGNGPGYKVVGGERENVSMVDYAHNNYQAQSAVPLRHETHGGEDVAIFAK
WHALE P.catodon XP_007111862.1                    DTDKKPFTAILYGNGPGYKVVGGERENVSMVDYAHNNYQAQSAVPLRHETHGGEDVAVFAK
ARMADILLO D.novemcinctus XP_004453534.1           DADKKPFTSILYGNGPGYKVVDGERENVSMVDYAHNNYQAQSAVPLRYETHGGEDVAVFAK
LESSSER HEDGEHOG E.telfairi XP_004705431.1        DTDKKPFTSILYGNGPGYKVVGGERENVSMVDYAHNNYQAQSAVPLRHETHGGEDVAVFAK
MOLE C.asiatica XP_006862403.1                    DTDKKPFTSILYGNGPGYKVVAGERENVSMVDYAHNNYQAQSAVPLRHETHGGEDVAVFAK
ELEPHANT L.africana XP_003413467.1                DTDKKPFTAILYGNGPGYKVVGGERENVSMVDYAHDNYQAQSAVPLRHETHGGEDVAVFAK
SHREW E.edwardii_XP_006883620.1                   DTDKKPFTAILYGNGPGYKVVGGERENVSMVDYAHNNYQAQSAVPLRHETHGGEDVAVFAK
AARDVARK O.a.afer XP_007933862.1                  DTDKKPFTAILYGNGPGYKVVGGERENVSMVDYAHNNYQAQSAVPLRHETHGGEDVAVFAK
RAT D.ordii XP_012890500.1                        DTDKKPFTAILYGNGPGYKVVGGERENVSMVDYAHNNYQAQSAVPLRHETHGGEDVAVFAK
MARMOT M.m.marmota XP_015341047.1                 DTDKKPFTAILYGNGPGYKVVDGERENVSMVDYAHNNYQAQSAVPLRHETHGGEDVAVFAK
SQUIRREL I.tridecemlineatus XP_005317611.1        DTDKKPFTAILYGNGPGYKVVDGERENVSMVDYAHNNYQAQSAVPLRHETHGGEDVAVFAK
JERBOA J.jaculus XP_004657609.1                   DTDKKPFTAILYGNGPGYKVVGGERENVSMVDYAHNNYQAQSAVPLRHETHGGEDVAVFAK
MOUSE M.musculus NP_001274101.1                   DTDKKPFTAILYGNGPGYKVVDGERENVSMVDYAHNNYQAQSAVPLRHETHGGEDVAVFAK
RAT R.norvegicus XP_003271616.1                   DTDKKPFTAILYGNGPGYKVVDGERENVSMVDYAHNNYQAQSAVPLRHETHGGEDVAVFAK
MOLE RAT_N.galili XP_008836842.1                  DTDKKPFTSILYGNGPGYKVVDGERENVSMVDYAHNNYQAQSAVPLRHETHGGEDVAVFAK
VOLE M.ochrogaster XP_013203781.1                 DTDKKPFTAILYGNGPGYKVVDGERENVSMVDYAHNNYQAQSAVPLRHETHGGEDVAVFAK
HAMSTER M.auratus XP_005081085.1                  DTDKKPFTAILYGNGPGYKVVDGERENVSMVDYAHNNYQAQSAVPLRHETHGGEDVAVFAK
HAMSTER C.griseus XP_003503700.1                  DTDKKPFTAILYGNGPGYKVVDGERENVSMVDYAHNNYQAQSAVPLRHETHGGEDVAIFAK
MOUSE P.m.bairdii XP_015854781.1                  DTDKKPFTAILYGNGPGYKVVDGERENVSMVDYAHNNYQAQSAVPLRHETHGGEDVAVFAK
GUINEA PIG_C.porcellus XP_003471396.1             DTDKKPFTAILYGNGPGYKVVGGERENVSTVDYAHNNYQAQSAVPLRHETHGGEDVAVFAK
DEGU O.degus XP_004637691.1                       DTDKKPFTAILYGNGPGYKVVGGERENVSMVDYAHNNYQAQSAVPLRHETHGGEDVAVFAK
CHINCHILLA C.lanigera XP_013375142.1              DTDKKPFTAILYGNGPGYKVVDGERENVSMVDYAHNNYQAQSAVPLRHETHGGEDVAVFAK
MOLE RAT_F.damarensis XP_010624322.1              DTDKKPFTAILYGNGPGYKVVGGERENVSMVDYAHNNYQAQSAVPLRHETHGGEDVAVFAK
NAKED MOLE_RAT_H.glaber XP_004850563.1            DTDKKPFTAILYGNGPGYKVVGGERENVSMVDYAHNNYQAQSAVPLRHETHGGEDVAVFAK
GALAGO O.garnettii XP_012657049.1                 DTDKKPFTAILYGNGPGYKVVDGERENVSTVDYAHNNYQAQSAVPLRYETHGGEDVAVFAK
RABBIT O.cuniculus XP_017201978.1                 DTDKKPFTAILYGNGPGYKVVGGERENVSMVDYAHNNYQAQSAVPLRHETHGGEDVAVFAK
LEMUR P.coquereli XP_012506383.1                  DTDKKPFTAILYGNGPGYKVVGGERENVSMVDYAHNNYQAQSAVPLRHETHGGEDVAVFAK
LEMUR M.murinus XP_012624158.1                    DTDKKPFTAILYGNGPGYKVVGGERENVSMVDYAHNNYQAQSAVPLRHETHGGEDVAVFAK
TARSIER C.syrichta_XP_008060913.1                 DTDKKPFTAILYGNGPGYKVVGGERENVSMVDYAHNNYQAQSAVPLRHETHGGEDVAVFAK
MONKEY S.b.boliviensis XP_003934953.1             DTDKKPFTAILYGNGPGYKVVGGERENVSMVDYAHNNYQAQSAVPLRHETHGGEDVAVFSK
MONKEY C.c.imitator XP_017362307.1                DTDKKPFTAILYGNGPGYKVVGGERENVSMVDYAHNNYQAQSAVPLRHETHGGEDVAVFSK
MONKEY A.nancymaae XP_012295593.1                 DTDKKPFTAILYGNGPGYKVVGGERENVSMVDYAHNNYQAQSAVPLRHETHGGEDVAVFSK
MONKEY C.jacchus NP_001171963.1                   DTDKKPFTAILYGNGPGYKVVGGERENVSMVDYAHNNYQAQSAVPLRHETHGGEDVAVFSK
MONKEY R.bieti XP_017742023.1                     DTDKKPFTAILYGNGPGYKVVGGERENVSMVDYAHNNYQAQSAVPLRHETHGGEDVAVFSK
MACAQUE M.fascicularis XP_005544582.1             DTDKKPFTAILYGNGPGYKVVGGERENVSMVDYAHNNYQAQSAVPLRHETHGGEDVAVFSK
MONKEY C.sabaeus XP_007978338.1                   DTDKKPFTAILYGNGPGYKVVGGERENVSMVDYAHNNYQAQSAVPLRHETHGGEDVAVFSK
BABOON P.anubis XP_003891323.1                    DTDKKPFTAILYGNGPGYKVVGGERENVSMVDYAHNNYQAQSAVPLRHETHGGEDVAVFSK
MACAQUE M.nemestrina XP_011741978.1               DTDKKPFTAILYGNGPGYKVVGGERENVSMVDYAHNNYQAQSAVPLRHETHGGEDVAVFSK
MACAQUE M.mulatta NP_001253798.1                  DTDKKPFTAILYGNGPGYKVVGGERENVSMVDYAHNNYQAQSAVPLRHETHGGEDVAVFSK
MONKEY C.a palliatus XP_011789361.1               DTDKKPFTAILYGNGPGYKVVGGERENVSMVDYAHNNYQAQSAVPLRHETHGGEDVAVFSK
MONKEY R.roxellana XP_010362643.1                 DTDKKPFTAILYGNGPGYKVVGGERENVSMVDYAHNNYQAQSAVPLRHETHGGEDVAVFSK
HUMAN H.sapiens NP_000469.3                       DTDKKPFTAILYGNGPGYKVVGGERENVSMVDYAHNNYQAQSAVPLRHETHGGEDVAVFSK
GIBBON N.leucogenys XP_003271616.1                DTDKKPFTAILYGNGPGYKVVGGERENVSMVDYAHNNYQAQSAVPLRHETHGGEDVAVFSK
GORILLA G.g.gorilla XP_004024892.1                DTDKKPFTAILYGNGPGYKVVGGERENVSMVDYAHNNYQAQSAVPLRHETHGGEDVAVFSK
CHIMPANZEE P.troglodytes XP_016811306.1           DTDKKPFTAILYGNGPGYKVVGGERENVSMVDYAHNNYQAQSAVPLRHETHGGEDVAVFSK
BONOBO P.paniscus XP_003813996.1                  DTDKKPFTAILYGNGPGYKVVGGERENVSMVDYAHNNYQAQSAVPLRHETHGGEDVAVFSK
ORANGUTAN P.abelii XP_002811389.1                 DTDKKPFTAILYGNGPGYKVVGGERENVSMVDYAHNNYQAQSAVPLRHETHGGEDVAVFSK
                                                  * *.  :*:* ******::.  . * *    .     * **:****  *****:***::::
```

Supplementary Figure 2. **Conservation of the Val^{434} position in 136 vertebrate species.** The protein sequence alignment spans from Asp^{404} to Lys^{464} (according to canine and human reference). The Val^{434} is highlighted, and yellow denotes those residues that differ from valine.

Supplementary Table 1. **Serum biochemistry in an affected and healthy 2-week-old littermate.**

Metabolite	Healthy puppy	Affected puppy	Reference range (adult dogs)	Unit
Albumin	23*	29	25-44	G/L
Alkaline phosphatase	98	10*	20-150	U/L
Alanine aminotransferase	18	14	10-118	U/L
Amylase	338	332	200-1200	U/L
Total bilirubin	8	4	2-10	UMOL/L
Blood urea nitrogen	5.7	10.0*	2.5-8.9	MMOL/L
Calcium	2.86	3.09*	2.15-2.95	MMOL/L
Phosphorus	3.12*	3.01*	0.94-2.13	MMOL/L
Creatinine	35	49	27-124	UMOL/L
Glucose	7.6*	6.9*	3.3-6.1	MMOL/L
Sodium	132*	138	138-160	MMOL/L
Potassium	5.4	5.0	3.7-5.8	MMOL/L
Total protein	40*	54*	54-82	G/L
Globulin	17*	24	23-52	G/L

*Out of reference range (only adult dog control ranges were available).

Supplementary Table 2. Samples used in NGS variant filtering.

Breed	WGS	WES	WGS + WES	Animals
Karelian Bear Dog, affected		1	1	1
Karelian Bear Dog, unaffected*	5	9	14	13
Affenpinscher	1		1	1
Afghan Hound		1	1	1
Airedale Terrier	2	6	8	8
Akita		3	3	3
Alaskan Husky mix	1		1	1
Alaskan Malamute	2	5	7	7
Alpine Dachsbracke	1		1	1
American Bulldog	2		2	2
American Hairless Terrier		5	5	5
American Staffordshire Terrier	1		1	1
Australian Cattle Dog	5		5	5
Australian Kelpie	5	12	17	13
Australian Shepherd	1		1	1
Australian Terrier	1	13	14	14
Basenji	6		6	6
Basset Griffon Vendeen	4		4	4
Basset Hound	3		3	3
Bavarian Hound	1		1	1
Beagle	6		6	6
Bearded Collie	11		11	11
Belgian Shepherd, gronendael		6	6	6
Belgian Shepherd, malinois	6	3	9	9
Belgian Shepherd, tervueren	2	7	9	9
Bichon Frisé	6	7	13	13
Black Russian Terrier	1		1	1
Border Collie	64	4	68	68
Border Terrier	1		1	1
Boston Terrier		1	1	1
Boxer	1	4	5	5
Bull Terrier	3		3	3
Bulldog		1	1	1
Bullmastiff	2		2	2
Cairn Terrier	2		2	2
Cane Corso	5		5	5
Cavalier King Charles Spaniel	3		3	3
Central Asian Shepherd Dog	1		1	1
Chihuahua	3	2	5	5
Chinese Crested Dog	1		1	1

Breed	WGS	WES	WGS + WES	Animals
Chinese indigenous dog	38		38	38
Chow Chow	1		1	1
Cocker Spaniel	1		1	1
Curly Coated Retriever	1		1	1
Dachshund	6	2	8	8
Dalmatian	3	4	7	7
Dandie Dinmont Terrier	1		1	1
Deerhound	1		1	1
Dobermann	5	9	14	14
Dogue de Bordeaux	6		6	6
Dutch Shepherd Dog	2		2	2
Elo	1		1	1
English Cocker Spaniel	2		2	2
English Springer Spaniel		1	1	1
Entelbuch Cattle Dog	8		8	8
Eurasier	2		2	2
Finnish Hound		3	3	3
Finnish Lapphund	2	6	8	6
Finnish Reindeer Herder		4	4	4
Finnish Spitz		9	9	9
Flanders Cattle Dog		2	2	2
Flat Coated Retriever	1		1	1
Foxterrier		4	4	4
French Bulldog	4	1	5	5
German Hunting Terrier	2		2	2
German Pinscher		4	4	4
German Pointing Dog	1	3	4	4
German Shepherd Dog	17	1	18	18
German Shepherd mix	3		3	3
Golden Retriever	8	3	11	11
Golden Retriever mix	1		1	1
Great Dane	1	16	17	17
Great Pyrenees	1		1	1
Greater Swiss Mountain Dog	6		6	6
Greyhound	1		1	1
Griffon	3		3	3
Grosspitz	1		1	1
Havanese	3		3	3
Heideterrier	1		1	1
Hovawart	1		1	1
Irish Soft Coated Wheaten Terrier	1	7	8	8
Irish Terrier	2		2	2

Breed	WGS	WES	WGS + WES	Animals
Irish Wolfhound	2		2	2
Italian Greyhound	2	1	3	3
Jack Russell Terrier	3		3	3
Japanese Chin	1		1	1
Kerry Blue Terrier	1		1	1
King Charles Spaniel		2	2	2
Kromfohrländer	1		1	1
Kuvasz		4	4	4
Labrador Retriever	42	10	52	52
Labrador Retriever mix	1		1	1
Lagotto Romagnolo	9	4	13	11
Lancashire Heeler		2	2	1
Landseer	2	2	4	4
Leonberger	54	4	58	57
Miniature Bullterrier	1		1	1
Miniature Schnauzer	25	2	27	27
Mixed Breed	1		1	1
Newfoundland	2	4	6	6
Norfolk Terrier		3	3	3
Norwegian Elkhound, grey		2	2	2
Norwegian Lundehund		1	1	1
Norwich Terrier	8	1	9	9
Nova Scotia Duck Tolling Retriever	1		1	1
Old English Sheepdog	1		1	1
Papillon	3		3	3
Parson Russell Terrier		4	4	4
Pomeranian	4		4	4
Poodle	22		22	22
Portuguese Water Dog	3	2	5	5
Pug	20		20	20
Pyrenean Sheepdog		1	1	1
Rhodesian Ridgeback	4	2	6	6
Rottweiler	2	3	5	5
Russian Black Terrier		4	4	4
Saint Bernard Dog	2		2	2
Saluki	5	2	7	7
Samoyed		1	1	1
Schnauzer	1	5	6	6
Shetland Sheepdog	2	3	5	5
Shih Tzu	4		4	4
Siberian Husky	3	2	5	5
Sloughi	3		3	3

Breed	WGS	WES	WGS + WES	Animals
Spanish Waterdog	1		1	1
Stabyhoun	5		5	5
Staffordshire Bull Terrier	1		1	1
Swedish Vallhund	3	2	5	5
Tibetan Mastiff	10	3	13	13
Tibetan Spaniel	1		1	1
Vizsla	2		2	2
Weimaraner	2		2	2
Welsh Corgi	2		2	2
Welsh Springer Spaniel	3		3	3
West Highland White Terrier	19	2	21	20
Whippet	4	3	7	7
White Swiss Shepherd Dog	3	2	5	5
Wolf	8		8	8
Yorkshire Terrier	67		67	67
Total samples in initial filtering	658	243	901	890
Grand total	663	252	915	903

NGS = Next generation sequencing, WGS = Whole genome sequence, WES = Whole exome sequence

*The unaffected KBDs were excluded from initial filtering

Supplementary Table 3. Prioritized homozygous variants in the affected dog.

Chr:position	REF > ALT	Variant type	Gene	Gene information	Variant details (ref. sequence)
1:47568351	C > T	splicing SNV	SYNJ2	Synaptojanin 2: inositol polyphosphate 5-phosphatase, associated to hearing loss in mice.	c.3423+11C>T (XM_014115768.2)
1:115347272	A > G	nonsynonymous SNV	ZNF527	Zinc finger protein 527: unknown function.	c.430A>G; p.R144G (XM_014115577.2)
1:115788268	C > T	nonsynonymous SNV	LOC100687072	ZFP30 zinc finger protein: unknown function.	c.505G>A; p.G169R (XM_014115673.2, low quality ref.)
1:116699524	G > GTAGTAGGCT	nonframeshift insertion	LOC102155339	IgGFc-binding protein-like: unknown function.	c.4112_4113insAGCCTACTA; p.G1371insGAYY (XM_022406688.1)
1:117146140	G > T	splicing SNV	DMKN	Dermokine: epidermal differentation, endosome trafficking, upregulated in inflammatory disease.	c.693+8G>T (XM_022403921.1)
1:117321334	G > A	nonsynonymous SNV	LSR	Lipolysis stimulated lipoprotein receptor: lipid metabolism, lipoprotein clearance.	c.1022C>T; p.P341L (XM_005616718.3, partial ref.)
2:77561953	A > C	nonsynonymous SNV	ALPL	Alkaline phosphatase, liver/bone/kidney: mutations cause hypophosphatasia in humans.	c.1301T>G; p.V434G (XM_005617214.3)
6:7824739	G > C	nonsynonymous SNV	CUX1	Cut like homeobox 1: transcription factor.	c.1602C>G; p.N534K (XM_005621000.3)
14:27216415	C > T	nonsynonymous SNV	VWDE	Von Willebrand factor D and EGF domains: unknown function.	c.4973G>A; p.G1658D (XM_022427414.1)
14:35556623	G > A	nonsynonymous SNV	DNAH11	Dynein axonemal heavy chain 11: mutations cause primary ciliary dyskinesia in humans.	c.5020G>A; p.G1674S (XM_022427467.1, low quality ref.)
15:63661871	G > A	nonsynonymous SNV	DDX60	DExD/H-box helicase 60: antiviral RNA helicase.	c.4840C>T; p.H1614Y (XM_532716.6)
18:49806934	T > G	nonsynonymous SNV	ALDH3B1	Aldehyde dehydrogenase 3 family member B1: aldehyde detoxification.	c.1094A>C; p.Y365S (XM_014121253.2)
18:55366074	G > T	nonsynonymous SNV	TMEM132A	Transmembrane protein 132A: unknown function.	c.1748C>A; p.P583H (XM_005631694.3)
21:32354052	C > A	nonsynonymous SNV	NRIP3	Nuclear receptor interacting protein 3: unknown function.	c.157G>T; p.G53C (XM_846198.4, low quality ref.)
25:50721076	C > T	nonsynonymous SNV	LOC102152569	Uncharacterised protein model: unknown function.	c.647C>T; p.P216L (XM_005635935.3)

REF = reference allele, ALT = alternative allele, SNV = single nucleotide variant

Supplementary Table 4. Pathogenicity prediction results.

Algorithm	Prediction	PredictSNP expected accuracy	Score	Patogenicity cutoff
PredictSNP consensus	Neutral	0,63	-	-
PredictSNP: MAPP	Neutral	0,65	-	-
PredictSNP: PhD-SNP	Deleterious	0,68	-	-
PredictSNP: PolyPhen-1	Neutral	0,67	-	-
PredictSNP: PolyPhen-2	Deleterious	0,40	-	-
PredictSNP: SIFT	Neutral	0,68	-	-
PredictSNP: SNAP	Neutral	0,58	-	-
PredictSNP: PANTHER	Deleterious	0,65	-	-
PROVEAN	Neutral	-	-2.446	<= -2.5
MutationTaster2*	Disease causing	-	0,999999992	1 is highest probability of accurate prediction

*The Mutation Taster2 algorithm was used with a human *ALPL* reference (NM_000478.5, NP_000469.3), and the other algorithms with the canine reference ((XM_005617214.3, XP_005617271.1)

Supplementary Video Legends

Supplementary Video 1. An affected puppy at 10 weeks of age (litter 1). The puppy is walking in a crouched position and showing both planti- and palmigrade gait (metatarsals and metacarpals on the ground).

Supplementary Video 2. An affected puppy at 2 weeks of age (litter 2). The affected puppy is having a seizure.
